# Oxidative Stress Marker Aberrations in Multiple Sclerosis: A Meta-Analysis Study

**DOI:** 10.3389/fnins.2020.00823

**Published:** 2020-08-26

**Authors:** Shu-Yao Zhang, Lue-Ning Gui, Yi-Ying Liu, Sha Shi, Yong Cheng

**Affiliations:** Key Laboratory of Ethnomedicine for Ministry of Education, Center on Translational Neuroscience, College of Life and Environmental Sciences, Minzu University of China, Beijing, China

**Keywords:** oxidative stress, multiple sclerosis, peripheral blood, cerebrospinal fluid, meta-analysis

## Abstract

Oxidative stress has been suggested to play a key role in multiple sclerosis (MS), but clinical data on oxidative stress markers in MS patients were inconsistent. This study sought to quantitatively summarize the data of oxidative stress markers in the blood and cerebrospinal fluid (CSF) of patients with MS in the literature. We conducted a systematic search of PubMed and Web of Science and included studies if they provided data on the concentrations of oxidative stress markers in the peripheral blood and CSF of MS patients and healthy control (HC) subjects. The systematic search resulted in the inclusion of 31 studies with 2,001 MS patients and 2,212 HC subjects for meta-analysis. Random-effects meta-analysis demonstrated that patients with MS had significantly increased concentrations of blood oxidative stress markers compared with HC subjects for malondialdehyde (MDA; Hedges' g, 2.252; 95% CI, 1.080 to 3.424; *p* < 0.001) and lipid hydroperoxide by tert-butyl hydroperoxide-initiated chemiluminescence (CL-LOOH; Hedges' g, 0.383; 95% CI, 0.065 to 0.702; *p* = 0.018). In contrast, concentrations of albumin (Hedges' g, −1.036; CI, −1.679 to −0.394; *p* = 0.002) were significantly decreased in MS patients when compared with those in HC subjects. However, the other analyzed blood oxidative stress markers did not show significant differences between cases and controls. Furthermore, this meta-analysis showed significant association between CSF MDA and MS (Hedges' g, 3.275; 95% CI, 0.859 to 5.691; *p* = 0.008). Taken together, our results revealed increased blood and CSF MDA and decreased blood albumin levels in patients with MS, strengthening the clinical evidence of increased oxidative stress in MS.

## Introduction

Multiple sclerosis (MS) is a disease characterized by inflammatory demyelinating lesions in the white matter of the central nervous system, which leads to the impairment of electrical signaling along an axon in the neurons (Hadzovic-Dzuvo et al., 2011). MS usually occurs in adults between the ages of 20 and 45 years, and it has a high disability rate (Goldenberg, 2012; Aboud and Schuster, 2019). The most common symptoms of the disease include spasticity, chronic pain, fatigue, motor and mobility disorders, and cognitive impairment (Aboud and Schuster, 2019). There is no reliable biomarker to predict the location and onset time of the MS, and currently available drugs only provide symptom alleviation (Goldenberg, 2012; Oliveira et al., 2019). Therefore, there is a need to understand the etiology of MS better and subsequently develop more effective drugs for the treatment of the disease.

The development of MS is considered to be an interaction of genetic predisposition, environmental factors, and aberrant immune response, but the precise etiology of the disease remains unknown (Chastain and Miller, 2012). Also, studies have suggested that oxidative stress may play an important role in the pathogenesis of MS (Schreibelt et al., 2007; van Horssen et al., 2011), as one of the common features in the brains of MS patients is the imbalance between oxidants and antioxidants (Wang et al., 2014; Pasquali et al., 2015; Trentini et al., 2017; De Riccardis et al., 2018), with increased concentrations of reactive oxygen species in cerebrospinal fluid (CSF) of MS patients (Acar et al., 2003; Gilgun-Sherki et al., 2004). Furthermore, clinical studies have shown increased oxidative stress in blood of MS patients, including dysregulated malondialdehyde (MDA) (Juybari et al., 2018), superoxide dismutase (SOD) (Tasset et al., 2012), and glutathione (GSH) (Gironi et al., 2014) levels in the patients. However, some studies showed the opposite results between MS patients and controls for SOD (De Riccardis et al., 2018) and GSH (Socha et al., 2014) levels.

Given the inconsistent findings on oxidative stress marker levels in MS patients, it is necessary to review the literature systematically to address this issue. Therefore, here, we performed a systematic search of studies reporting blood and CSF oxidative stress marker levels in patients with MS and quantitatively summarized the oxidative stress marker data with a meta-analytic technique.

## Methods

The meta-analyses implemented in this study conform to the instructions that are recommended by the Preferred Reporting Items for Systematic Reviews and Meta-Analysis statement (Moher et al., 2009). Furthermore, we followed the methods of (Wei et al., 2018).

### Search Strategy and Study Selection

A systematic review of peer-reviewed English articles from the databases of PubMed and Web of Science was performed from September 2019 to October 2019. The search term used for the database search was: multiple sclerosis and (oxidative stress or superoxide dismutase or malondialdehyde or glutathione or total antioxidant status or total oxidation state or C-reactive protein or triglycerides or albumin or advanced oxidation protein products or catalase or hydroxyguanosine or uric acid or ceruloplasmin or transferrin or low-density lipoprotein or cholesterol), without year restriction. Clinical studies were included if they reported data on circulating oxidative stress marker concentrations in MS patients and healthy control (HC) subjects. Exclusion criteria were (1) no necessary concentration data; (2) oxidative stress markers were measured in animal models; (3) no HC subjects; (4) samples were overlapping with other studies; (5) *in vitro* data; (6) patients had serious complications; and (7) individual oxidative stress marker was assessed in <3 studies.

### Data Extraction

Data of mean oxidative stress marker concentration, standard deviation (SD), and sample size were extracted to calculate the effective size (ES) for meta-analysis. We also extracted data on age, sex, disease duration, BMI, expanded disability status scale, and sampling sources for potential heterogeneity analysis among studies (Supplementary Table 1).

### Statistical Analysis

All statistical analysis was achieved by comprehensive meta-analysis software 2. ES was mainly generated by sample size, mean oxidative stress marker concentration, and SD. The standardized mean difference of oxidative stress marker concentration between MS patients and HC subjects was calculated as ES and converted into Hedge's g statistic to provide an unbiased adjusted ES for sample size. A random-effects model was used in the meta-analysis because we assumed that between- and within-study heterogeneity affected the true ES. Sensitivity analysis was performed to test the robustness of the results of meta-analysis, which is achieved by excluding one study at a time to perform a meta-analysis.

Between-study heterogeneity was assessed by the Cochrane Q test and *I*^2^ statistic (Qin et al., 2017), and *P* < 0.10 was considered statistically significant for the Cochrane Q test. The inconsistency across studies was determined by *I*^2^ index to evaluate the impact of heterogeneity, and *I*^2^ values of 0.25, 0.5, and 0.75 indicate small, moderate, high levels of between-study heterogeneity, respectively. Unrestricted maximum-likelihood random-effects meta-regression analyses of ES were performed to test whether sample size, patient age, and sex (proportion of male individuals), and disease duration had moderating effects on the outcomes of the meta-analysis. Potential publication bias was determined by Egger's test.

*P* < 0.05 was considered statistically significant in the meta-analysis except for where noted.

## Results

**Figure 1:**
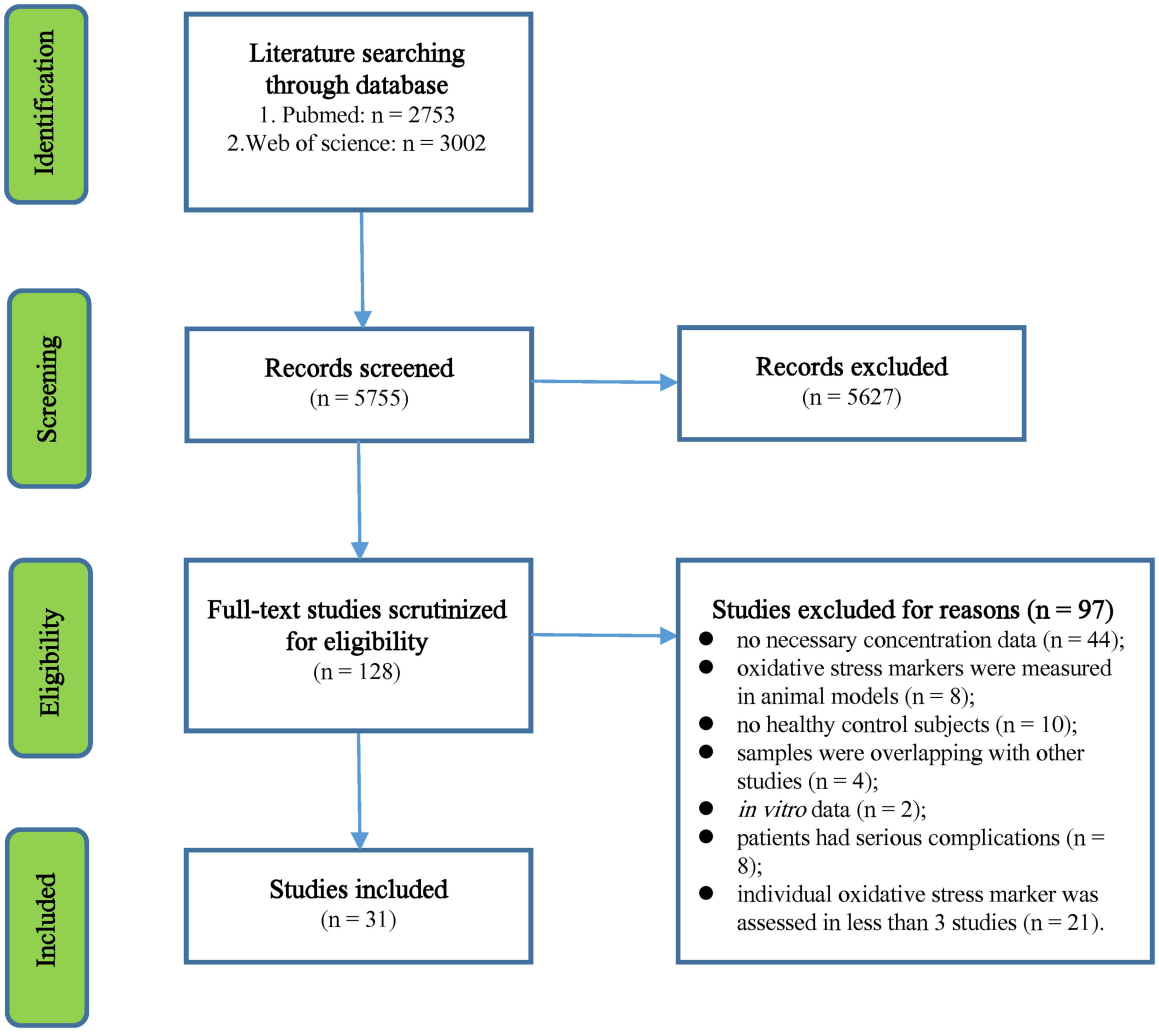
Preferred Reporting Items for Systematic Reviews and Meta-Analysis flowchart of the literature search.

The initial search yielded 2,753 records from the PubMed database and 3,002 records from Web of Science. We performed a preliminary screening of the titles and abstracts of the 5,755 records, and 128 articles that were relevant to this study were selected for full-text scrutiny. After full-text scrutiny, 97 studies were excluded because (1) no necessary concentration data (*n* = 44), (2) oxidative stress markers were measured in animal models (*n* = 8), (3) no HC subjects (*n* = 10), (4) samples were overlapping with other studies (*n* = 4), (5) *in vitro* data (*n* = 2); (6) patients had serious complications (*n* = 8), and (7) individual oxidative stress marker was assessed in <3 studies (*n* = 21). Therefore, a total of 31 studies (Jimenez-Jimenez et al., 1998; Keles et al., 2001; Ghabaee et al., 2010; Fjeldstad et al., 2011; Hadzovic-Dzuvo et al., 2011; Miller et al., 2011; Tavazzi et al., 2011; Acar et al., 2012; Oliveira et al., 2012, 2017a,b; Tasset et al., 2012; Ashtari et al., 2013; Kirbas et al., 2013; Ljubisavljevic et al., 2013; Aydin et al., 2014, 2015; Gironi et al., 2014; Polachini et al., 2014; Socha et al., 2014, 2017; Wang et al., 2014; Yousefi et al., 2014; Moccia et al., 2015; Bartova et al., 2016; Kallaur et al., 2016; Bystricka et al., 2017; Delgado-Roche et al., 2017; De Riccardis et al., 2018; Juybari et al., 2018; Armon-Omer et al., 2019), including 2,001 MS patients and 2,212 HC subjects, were included in the meta-analysis (Flowchart see Figure 1).

### Association of Multiple Sclerosis With Blood Oxidative Stress Markers

**Figure 2:**
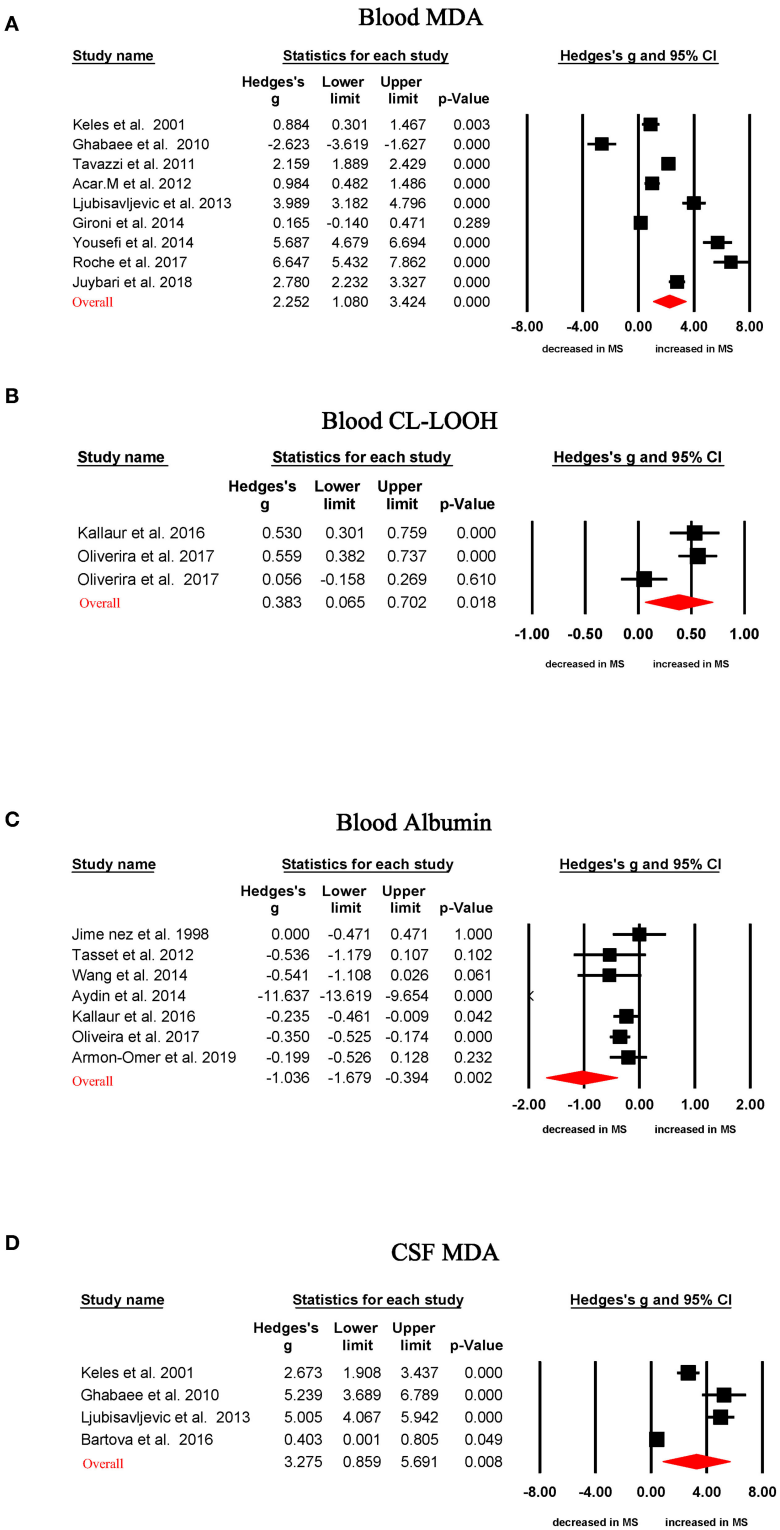
Studies of circulating MDA, CL-LOOH, and albumin in multiple sclerosis. Forest plot displaying random-effects meta-analysis results between blood MDA **(A)**, blood CL-LOOH **(B)**, blood albumin **(C)**, CSF MDA **(D)**, and multiple sclerosis. Standardized mean difference (Hedges g) on the horizontal axis; positive values denote higher in patients with multiple sclerosis; negative values denote higher in healthy control subjects. The diamond denotes pooled effective size (Hedges' g). MDA, malondialdehyde; CL-LOOH, lipid hydroperoxide by tert-butyl hydroperoxide-initiated chemiluminescence; CSF, cerebrospinal fluid.

Random-effects meta-analysis demonstrated that patients with MS had significantly increased blood oxidative stress marker levels compared with HC subjects for MDA (Hedges' g, 2.252; 95% CI, 1.080 to 3.424; *p* < 0.001) and lipid hydroperoxide by tert-butyl hydroperoxide-initiated chemiluminescence (CL-LOOH; Hedges' g, 0.383; 95% CI, 0.065–0.702; *p* = 0.018), as shown in Table 1, Figures 2A,B. In contrast, concentrations of albumin (Hedges' g, −1.036; CI, −1.679 to −0.394; *p* = 0.002) were significantly lower in MS patients than that of HC subjects (Table 1, Figure 2C). However, blood levels of GSH, SOD, total antioxidant status, total oxidative status, C-reactive protein, copper, cholesterol, uric acid, and advanced oxidation protein product did not show significant differences between MS patients and HC subjects (Table 1).

**Table 1:** Summary of comparative outcomes for measurements of blood and CSF marker levels.

**OS factors**	**No. of studies**	**No. with MS/HC**	**Main effect**			**Heterogeneity**				**Publication bias**	
			**Hedge's g (95% CI)**	**Z score**	***P*-value**	**Q statistic**	**Df**	***P*-value**	***I*^**2**^ statistic**	**Egger intercept**	***P*-value**
**CSF**											
MDA	4	157/55	3.275 (0.859 to 5.691)	2.657	0.008	113.388	3	0.000	97.354	11.17546	0.06674
**BLOOD**											
MDA	9	508/455	2.252 (1.080 to 3.424)	3.766	0.000	362.466	8	0.000	97.793.	4.66669	0.36864
CL-LOOH	3	594/912	0.383 (0.065 to 0.702)	2.362	0.018	14.374	2	0.001	86.086	−7.89791	0.74166
Albumin	7	554/683	−1.036 (−1.679 to −0.394)	−3.163	0.002	129.113	6	0.000	95.353	−5.12036	0.13671
AOPP	3	501/716	−0.074 (−0.386 to 0.238)	−0.467	0.640	13.976	2	0.001	85.689	12.84954	0.52538
TOS	3	100/97	0.615 (−0.158 to 1.389)	1.559	0.119	14.416	2	0.001	86.127	31.19051	0.24010
Copper	3	113/99	−5.114 (−11.285 to 1.056)	−1.625	0.104	154.921	2	0.000	98.709	−13.50436	0.23795
Cholesterol	9	371/536	0.358 (−0.092 to 0.808)	1.558	0.119	73.599	8	0.000	89.130	2.30119	0.42661
SOD	6	154/164	−1.418 (−3.332 to 0.496)	−1.452	0.146	206.596	5	0.000	97.580	−4.42659	0.71014
TAS	5	260/194	−0.281 (−1.699 to 1.136)	−0.389	0.697	143.068	4	0.000	97.204	5.34355	0.62950
CRP	5	429/541	−0.499 (−1.045 to 0.048)	−1.788	0.074	42.513	4	0.000	90.591	−3.13185	0.28983
GSH	6	285/269	−0.707 (−1.674 to 0.259)	−1.434	0.152	121.054	5	0.000	95.870	−2.05131	0.76032
UA	13	1,072/893	−0.126 (−0.401 to 0.148)	−0.901	0.367	88.776	12	0.000	86.483	−1.35441	0.47419

*df, degrees of freedom; MDA, malondialdehyde; CL-LOOH, lipid hydroperoxide by tert-butyl hydroperoxide-initiated chemiluminescence; AOPP, advanced oxidation protein product; TOS, total oxidative status; SOD, superoxide dismutase; TAS, total antioxidant status; CRP, total oxidative status; GSH, glutathione; UA, uric acid. CSF, cerebrospinal fluid*.

### Association of Multiple Sclerosis With Cerebrospinal Fluid Oxidative Stress Markers

Random-effects meta-analysis showed CSF MDA levels were significantly increased in MS patients when compared with those in HC subjects (Table 1, Figure 2D; Hedges' g, 3.275; 95% CI, 0.859 to 5.691; *p* = 0.008). However, there were not enough studies to perform a meta-analysis for other CSF oxidative stress markers.

### Investigation of Heterogeneity

All of the 13 oxidative stress markers (12 in blood and 1 in CSF) analyzed in the meta-analysis showed high levels of between-study heterogeneities (Table 1). Of the oxidative stress markers that were significantly associated with MS, only blood MDA had a relatively large number of studies. We, therefore, tried to perform meta-regression analyses to test whether potential confounders, including age and sex, had moderating effects on blood MDA levels in MS patients, given that information on disease severity and BMI on the patients were limited.

**Figure 3:**
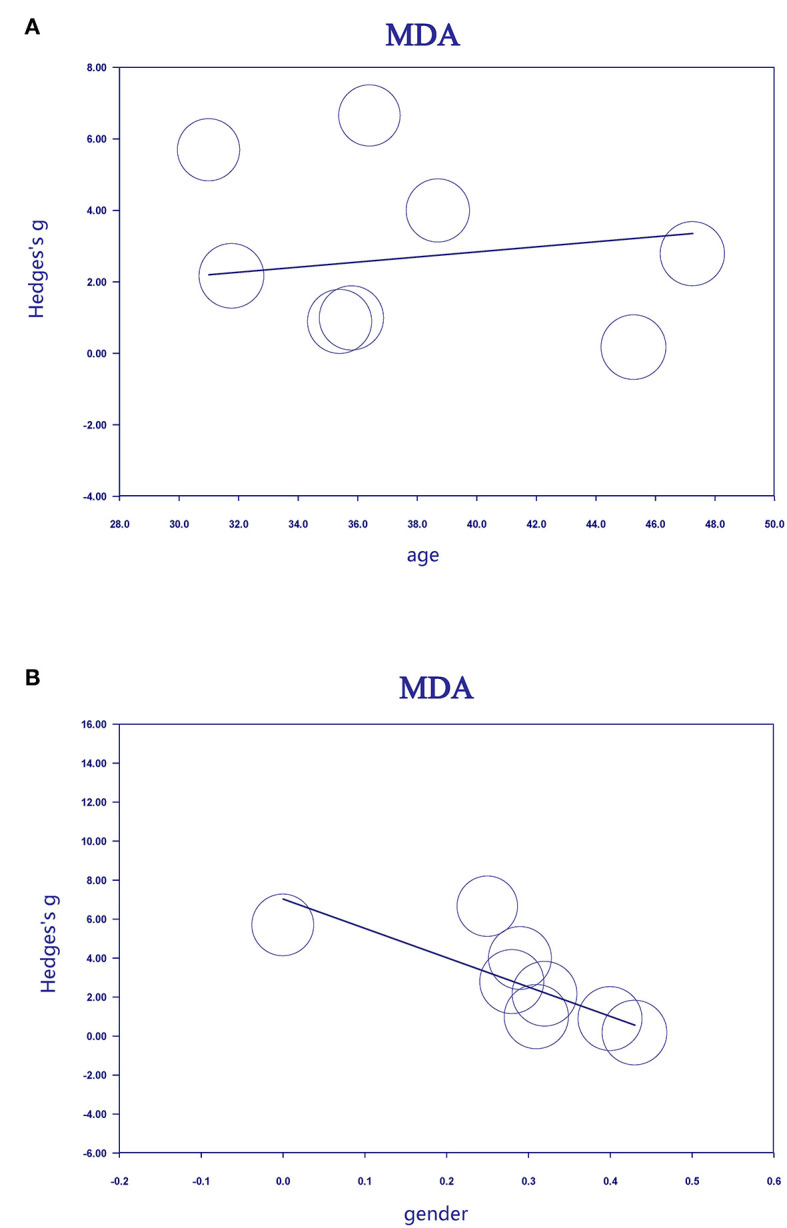
Association between age **(A)**, sex **(B)**, and effective size (Hedges' g) for blood MDA. MDA, malondialdehyde.

Meta-regression analyses showed that age was positively associated with ES for studies measuring MDA levels in MS patients (Figure 3A, regression coefficient [SE], 0.1861 [0.0436]; 95% CI, 0.1007 to 0.2715; *P* < 0.001). In contrast, sex was negatively associated (Figure 3B, regression coefficient [SE], −15.0457 [5.3227]; 95% CI, −25.4781 to −4.6133; *P* = 0.0047) with ES for studies measuring MDA levels in MS patients.

Sensitivity analysis showed that no single study affected the significant difference in blood MDA levels between MS patients and HC subjects.

The Egger's test suggested that there was no significant risk of publication bias for the included studies analyzing MDA levels (Table 1).

## Discussion

To the best of our knowledge, this is the first systematic review and meta-analysis on peripheral blood and CSF oxidative stress marker levels in patients with MS. The meta-analysis included 31 articles with 2,001 MS patients and 2,212 HC subjects and reported that CL-LOOH and MDA levels were significantly increased in patients with MS when compared with those in controls. Furthermore, CSF MDA levels were found to be elevated in MS patients. In contrast, albumin levels were significantly decreased in patients with MS when compared with those in controls. Sensitivity analysis demonstrated that the significant association of MS with MDA was not influenced by any single study, suggesting the robustness of the outcomes of the meta-analysis. Although results from previous studies were inconsistent for oxidative stress marker data in MS, the present meta-analysis provides strong clinical evidence of increased oxidative stress in patients with MS. Our study thus clarified the circulating oxidative stress marker profile in patients with MS and may enhance our understanding of the etiology of MS.

In general, oxidative stress is caused by an imbalance between free radical production and antioxidant defensive system (Supplementary Figure 1). Increased free radicals, including reactive oxygen species and reactive nitrogen species, can lead to lipid and protein damages through peroxidation and nitration processes (Adamczyk and Adamczyk-Sowa, 2016). Both MDA and CL-LOOH are considered to be markers for lipid damage in the body, and MDA can cause cross-linking polymerization of proteins, nucleic acids, and other life macromolecules, leading to cytotoxicity. Our meta-analysis showed blood MDA, and CL-LOOH levels were significantly increased in patients with MS, suggesting that lipid damage in patients with MS may contribute to neurodegeneration in the disease. These results are consistent with a previous study suggesting that lipophilic antioxidant deficiency in the blood of MS patients contributed to the reduced reparative demyelinating processes and promoted neurodegeneration (Kuracka et al., 2014).

Although the meta-analysis provided strong clinical evidence of increased oxidative stress in patients with MS, whether oxidative stress is involved in the pathogenesis of MS is unclear. However, the hypothesis that oxidative stress contributes to the progress of MS is plausible, given the considering evidence showing the beneficial effects of antioxidants on MS patients (Adamczyk and Adamczyk-Sowa, 2016). Indeed, one study reported that melatonin reduced oxidative stress, included increased MDA levels, and increased SOD and GSH peroxidase activities, suggesting a positive effect of melatonin on the progression of the severe form of MS (Miller et al., 2013). Additionally, a randomized, double-blinded, placebo-controlled trial showed that supplementation of coenzyme Q10 in relapsing–remitting MS patients increased plasma SOD activity and reduced MDA levels in the patients (Sanoobar et al., 2013). Direct evidence of functional involvement of oxidative stress in MS pathogenesis comes from animal models of MS. Ghaffari et al. utilized a toxic model of MS by intrahippocampal injection of ethidium bromide in rats and showed that saffron extract treatment improved learning and memory in the experimental model of MS. The improved cognition by saffron extract treatment in animals was accompanied by the restoration of antioxidant status, including reduced MDA levels in the hippocampus (Ghaffari et al., 2015). In another animal model of MS, Wang et al. used a natural, endogenous antioxidant-α-lipoic acid to treat experimental autoimmune encephalomyelitis mice and showed that α-lipoic reduced disease severity by inhibition of infiltration of inflammatory cells into the central nervous system (Wang et al., 2013). Therefore, these results, together with the data from the present meta-analysis, suggest that oxidative stress is a promising target for MS treatment.

In addition to the increased oxidative stress found in MS patients, oxidative stress is suggested to be involved in other neurodegenerative diseases, such as Alzheimer's disease (AD), Parkinson's disease (PD), and amyotrophic lateral sclerosis (ALS). Although clinical data on oxidative stress markers levels in patients with AD, PD, and ALS were inconsistent in the literature, previous meta-analyses demonstrated patients with AD (Schrag et al., 2013), PD (Wei et al., 2018), and ALS (Wang et al., 2019) were accompanied by increased peripheral blood MDA levels, similar to the heightened blood MDA levels in MS patients found in the present meta-analysis. These results suggest that lipid damage might be a common phenomenon for neurodegenerative diseases. However, a limitation of this meta-analysis is that most oxidative stress markers were assessed in a small number of studies; these include SOD and GSH, which make it difficult to observe significant associations of MS with these oxidative stress markers, therefore, precludes us from comparing MS with other neurodegenerative diseases for most of the oxidative stress markers.

The second limitation of the meta-analysis is that only a few CSF studies were included; therefore, it is difficult to compare blood with CSF on oxidative stress marker levels. However, the meta-analysis found that blood and CSF MDA levels were consistently increased in MS patients. Therefore, we hypothesize good parallels between central and peripheral on the changes of oxidative stress marker levels in MS patients, and future studies are necessary to confirm this hypothesis. Third, due to the limited number of studies analyzing oxidative stress marker levels in different MS subtypes, whether oxidative stress marker levels were differentially changed in different disease stages is unclear, and further longitudinal studies are required to address the issue. The last limitation is that we only include English articles in the meta-analysis, which may create potential publication bias. However, given the small number of non-English articles in this field, non-English articles are not likely to significantly affect the outcomes of the meta-analysis.

## Data Availability Statement

The data used to support the findings of this study are included in the article.

## Author Contributions

YC and SS conceived and designed the study. S-YZ and L-NG collected the data. S-YZ, L-NG, Y-YL, SS, and YC analyzed and interpreted the data. S-YZ drafted the manuscript with critical revisions from all the authors.

## Conflict of Interest

The authors declare that the research was conducted in the absence of any commercial or financial relationships that could be construed as a potential conflict of interest.
