# Supplementary material for: Oxidative Stress Marker Aberrations in Multiple Sclerosis: A Meta-Analysis Study
Source: Front Neurosci. 2020 Aug 26;14:823. doi: 10.3389/fnins.2020.00823 (PMC7479227; doi:10.3389/fnins.2020.00823)
Supplement: Supplementary file 1 [file Table_1.pdf]

Supplementary Table 1: Characteristics of included studies measuring peripheral blood and CSF biomarker concentrations

Author, year	OS factors Measured	Country	Samples (MS/HC)	Gender	Age	Sample Source	Duration	BMI(MS/HC)	EDSS	Subtype
Jime nez et al. 1998	Albumin	Spain	36/32	0.338	31.8	serum	NA	NA	NA	NA
Keles et al. 2001	MDA	Turkey	30/20	0.4	35.4	CSF	NA	NA	NA	NA
Keles et al. 2001	MDA	Turkey	30/20	0.4	35.4	serum	NA	NA	NA	NA
Ghabaee et al. 2010	MDA, catalase	Iran	13/15	NA	NA	CSF	NA	NA	NA	NA
Ghabaee et al. 2010	MDA, catalase	Iran	13/15	NA	NA	serum	NA	NA	NA	NA
Fjeldstad et al. 2011	CRP	USA	44082	0	NA	serum	NA	NA	NA	NA
Hadžović-Džuvo et al. 2011	TAS	Bosnia and Herzegovina	33/24	NA	3.24	plasma	NA	NA	NA	NA
Miller et al. 2011	TAS, isoprostane	Poland	44130	0.39	47.53	plasma	10.0 ±6.5	NA	6.5 ±2.0	SPMS

Author, year	OS factors Measured	Country	Samples (MS/HC)	Gender	Age	Sample Source	Duration	BMI(MS/HC)	EDSS	Subtype
Tavazzi et al. 2011	MDA	Italy	170/163	0.32	31.77	serum	13.5±5.22	NA	3.26 ± 2.29	NA
Oliveira et al. 2012	UA	Brazil	91/196	0.38	37.61	plasma	NA	24.38/24.09	NA	RRMS
Tasset et al. 2012	GSH, CRP, albumin, cholesterol, UA	Spain	24/15	0.31	40.77	plasma	8.4±8.8	NA	NA	NA
Acar.M et al. 2012	SOD, MDA, TOS	Turkey	35/32	0.31	35.8	serum	6.22	NA	NA	NA
Kirbas et al. 2013	Cholesterol, TOS, TAS	Turkey	30/30	0.33	29.05	serum	NA	24.9 ± 4.1 /25.8 ± 4.7	NA	RRMS
Ashtari et al. 2013	UA	Iran	130/50	0.19	31.92	serum	5.4±1.8	NA	NA	NA
Ljubisavljevic et al. 2013	AOPP ,MDA, catalase, isoprostane	Serbia	57/20	0.29	38.7	CSF	7	NA	NA	NA
Ljubisavljevic et al.2013	AOPP, MDA, catalase, isoprostane	Serbia	57/20	0.29	38.7	serum	7	NA	NA	NA
Polachini et al. 2014	UA	Brazil	29/29	0.33	37.95	serum	9.65 ±1.7	NA	NA	NA

Author, year	OS factors Measured	Country	Samples (MS/HC)	Gender	Age	Sample Source	Duration	BMI(MS/HC)	EDSS	Subtype
Socha et al. 2014	GSH,TAS	Poland	101/63	0.35	40.96	serum	NA	NA	NA	NA
Wang et al. 2014	CRP, albumin, cholesterol, UA	China	30/20	0.3	40.4	serum	43840	NA	16	NA
Gironi et al. 2014	GSH,MDA	Italy	87/77	0.43	45.27	serum	NA	NA	NA	NA
Yousefi et al. 2014	MDA	Iran	38/38	0	31	serum	NA	NA	NA	NA
Aydin et al. 2014	TOS, Albumin, TAS	Turkey	35/35	0.286	38	serum	NA	NA	NA	NA
Aydin et al. 2015	UA, TOS, TAS	Turkey	35/35	0.4	38	plasma	6.8 ±5.4	26 ± 5.3/25 ± 4.2	1.8 (1–5)	NA
Moccia et al. 2015	UA	Italy	362/181	0.41	42.3	plasma	14.5±9.5	NA	3.9±1.4	NA
Kallaur et al. 2016	CL-LOOH, CRP, ferritin, albumin, TRAP	Brazil	108/249	0.29	37.8	serum	NA	25.2±5.1 /25.1±4.4	NA	NA
Bartova et al. 2016	MDA	Slovakia	57	0.46	49	CSF	NA	NA	NA	NA

Author, year	OS factors Measured	Country	Samples (MS/HC)	Gender	Age	Sample Source	Duration	BMI(MS/HC)	EDSS	Subtype
Oliveira et al. 2017	CL-LOOH,AOPP，NO	Brazil	137/218	0.32	39.97	serum	8.04±7.20	25.46±4.54/24.68±5.46	3.28±2.35	NA
Socha et al. 2017	Cooper	Poland	101/68	0.34	40.58	serum	NA	NA	3.34 ±1.5	NA
Oliveira et al. 2017	CL-LOOH, AOPP, CRP, ferritin, albumin, TRAP, NO	Brazil	258/249	0.29	39.95	serum	NA	25.09±5.1/25.09±4.4	NA	MS
Bystrická et al. 2017	GSH	Slovakia	37/37	0.22	39	serum	NA	NA	2 ±1	RRMS
Bystrická et al. 2017	GSH	Slovakia	8/37	0.22	41.71	serum	NA	NA	4 ±1	SPMS
Roche et al. 2017	SOD,MDA,AOPP,GSH	Jordan	28/40	0.25	36.4	serum	NA	NA	NA	NA
De Riccardis et al. 2018	Cooper, SOD	Italy	38/39	0.39	37.33	serum	NA	NA	NA	NA
De Riccardis et al. 2018	Cooper, SOD	Italy	19/18	0.43	35.22	CSF	NA	NA	NA	NA
Juybari et al. 2018	MDA	Iran	50/50	0.28	47.45	serum	5 ±2.5	26.1±4.9	3.8	RRMS

Author, year	OS factors Measured	Country	Samples (MS/HC)	Gender	Age	Sample Source	Duration	BMI(MS/HC)	EDSS	Subtype
Armon-Omer et al. 2019	Albumin	Israel	63/83	0.377	42.369	serum	NA	25	NA	NA

Abbreviations: *OS, Oxidative stress; MDA, malondialdehyde; CL-LOOH, lipid hydroperoxide by tert-butyl hydroperoxide-initiated chemiluminescence; AOPP, advanced oxidation protein product; TOS, total oxidative status; SOD, superoxide dismutase; TAS, total antioxidant status; CRP, total oxidative status; GSH, glutathione; TRAP, total radical-trapping antioxidant parameter; NO, nitric oxide; UA, uric acid; BMI, body mass index; EDSS, expanded disability status scale; NA, not available; MS, multiple sclerosis; RRMS, relapsing remitting multiple sclerosis; SPMS, secondary-progressive multiple sclerosis; HC, healthy control.*
