# Supplementary material for: Oxidative Stress Marker Aberrations in Multiple Sclerosis: A Meta-Analysis Study
Source: Front Neurosci. 2020 Aug 26;14:823. doi: 10.3389/fnins.2020.00823 (PMC7479227; doi:10.3389/fnins.2020.00823)
Supplement: Supplementary file 2 [file Image_1.pdf]

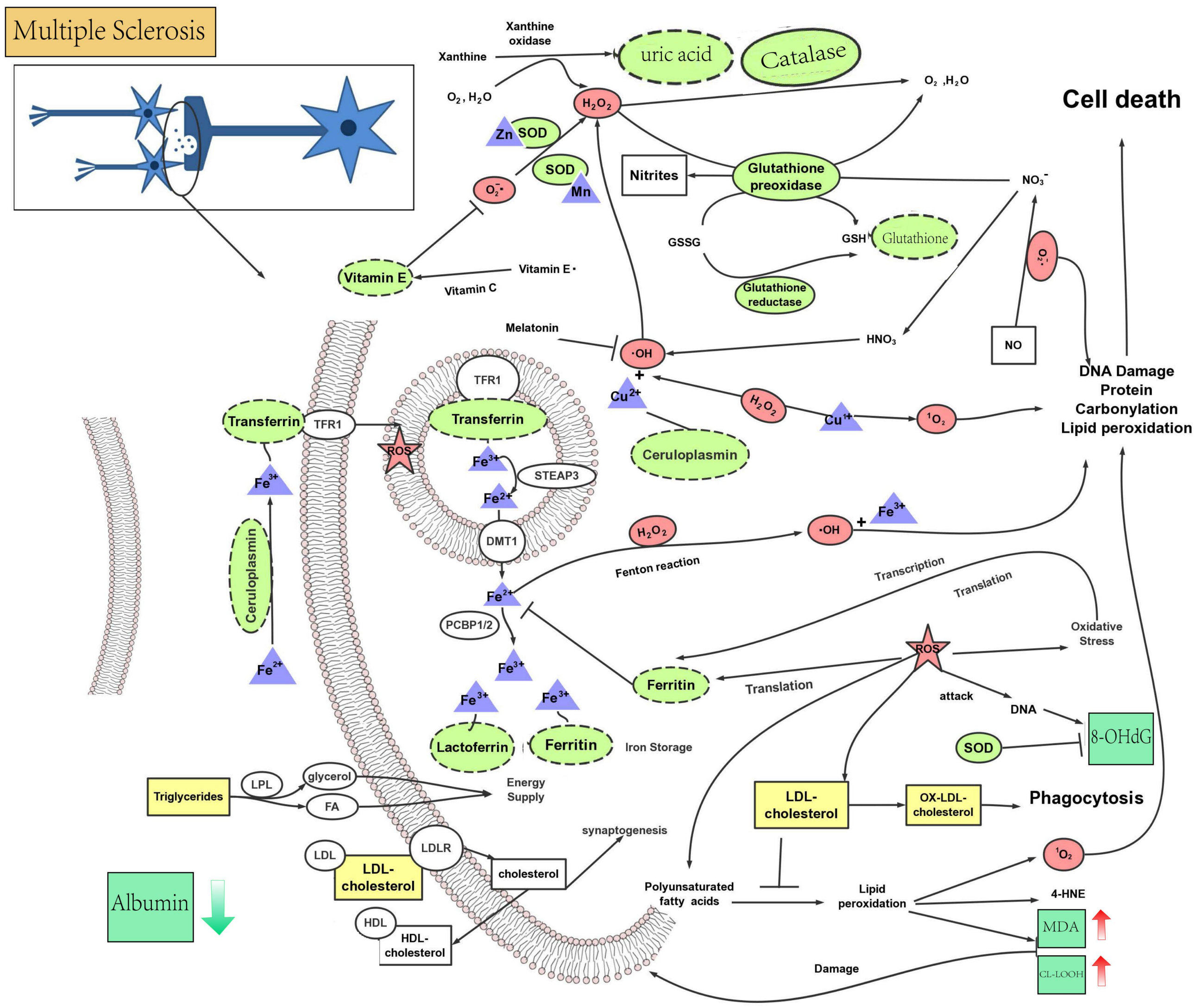

Supplementary Figure 1. Potential relationships between free radicals and antioxidant defenses.
